# Nuclear Localization Marker of FOXO3a: Can it be Used to Predict Doxorubicin Response?

**DOI:** 10.3389/fonc.2013.00149

**Published:** 2013-06-05

**Authors:** Chun Gong, Ui-Soon Khoo

**Affiliations:** ^1^Department of Pathology, Li Ka Shing Faculty of Medicine, The University of Hong KongHong Kong, China

Forkhead box O (FOXO) proteins, are a subgroup of transcription factors characterized by a conserved DNA-binding forkhead domain. FOXO3a is one of the members of this subfamily which activate or repress multiple genes involved in cell cycle regulation, apoptosis, DNA damage repair, protection against oxidative stress, and metabolism. FOXOs are regulated by a broad variety of stimuli which control FOXO protein expression, subcellular localization, DNA binding, and transcriptional activity. The biological activity of FOXO proteins are primarily regulated by post-translational modifications, including phosphorylation, acetylation, and ubiquitination.

The PI3K/Akt (PKB) pathway is one of the first and potentially most important kinase pathways shown to regulate FOXO3a activity (Paradis and Ruvkun, 1998). Phosphorylation of FOXO3a by Akt results in cytoplasmic accumulation and its subsequent degradation. ERK (Biggs et al., 1999; Yang et al., 2008) and JNK (Sunters et al., 2006), two of the three canonical MAPK pathways (ERK, p38, and JNK), have also been shown to activate FOXO3a activity, but nothing had previously been known with regard the p38 MAPK signaling pathway in relation to FOXO protein regulation. Phosphorylations at most of the known sites of FOXO3a lead to its translocation into cytoplasm and inactivation (Yang and Hung, 2009). Based on this, functional studies of FOXO3a could only examine its activity indirectly through checking the expression of its inactive form.

FOXO3a has been shown to play an important role in mediating the cytotoxic effects of chemotherapeutic drugs such as doxorubicin (Hui et al., 2008a,b). It has been observed that acquisition of chemoresistance correlated unexpectedly with increased expression and nuclear accumulation of FOXO3a in leukemic cells (Hui et al., 2008b) and breast cancer cells (Chen et al., 2010). Indeed, examination in breast cancer tissue microarrays showed sustained nuclear FOXO3a was associated with poor prognosis (Chen et al., 2010). It is speculated that FOXO3a engages in a feedback mechanism whereby sustained FOXO3a activation can enhance hyperactivation of the PI3K/Akt pathway (Gomes et al., 2008).

Lam and co-workers in the captioned article, report a novel phosphorylation site of FOXO3a which appears responsible for its nuclear relocation under the action of doxorubicin (Ho et al., 2012). They observed FOXO3a nuclear relocation as well as induction of p38 expression following doxorubicin treatment and hypothesized that p38 could directly regulate FOXO3a and provided concrete evidence to support their speculation. They elegantly demonstrated that p38 not only complexes with FOXO3a with or without doxorubicin treatment, but also directly phosphorylates FOXO3a at a novel site, Ser-7. Phosphorylation of FOXO3a at Ser-7 is a key for its relocation to nucleus, as a Ser-7 phosphorylation-deficient mutant could not be localized to nucleus when treated with doxorubicin (Ho et al., 2012).

The involvement of the stress activated kinase p38 targeting FOXO3a opens further horizons for investigation with regard to other p38 phosphorylation sites that may contribute toward FOXO3a nuclear relocalization. Indeed additional p38 phosphorylation sites were identified in this study which are also targeted by JNK and ERK. Furthermore p38 phosphorylation may also have other biological functions not yet identified. In terms of anti-cancer therapeutics, due to the potent antitumor activity of FOXO3a, it has been suggested that drugs that activate FOXO3a may be used in combination with other therapeutic agents to sensitize tumor cells. The dependence of chemotherapeutic-induced cell death on p38 activation is well documented.

Ser-7 is the first reported site at which phosphorylation can lead to the nuclear localization of FOXO3a. It could serve as a marker for nuclear FOXO3a as suggested by the authors. However, caution should be taken when using it as a marker for active FOXO3a, for though it is generally acknowledged that nuclear FOXO3a is active, this should not be taken for granted. The authors did not show directly that the phosphorylation at Ser-7 was a causal event of FOXO3a activation. As shown in their supplementary data, over-expression of Ser-7 phosphorylation-deficient mutants could still induce the activation of FOXO3a and the expression of its target genes (Ho et al., 2012). Hence the functional importance of Ser-7 phosphorylated FOXO3a remains to be further investigated.

This article mainly used MCF7, a doxorubicin-sensitive breast cancer cell line, as a model. However, the functions of FOXO3a have been indicated to change from pro-apoptotic to pro-survival in cell lines with acquire resistance to doxorubicin (Hui et al., 2008a; Wilson et al., 2011). Therefore, besides fully exploring the functions of nuclear Ser-7 phosphorylated FOXO3a in the current cell line model, it would also be interesting to study the role of FOXO3a phosphorylation at Ser-7 in acquired doxorubicin resistance. It was noted that Ser-7 phosphorylation by doxorubicin also occurs in prostate cancer cell lines, and was suggested that it may be a universal event. As mentioned earlier, nuclear accumulation of FOXO3a and hyper-active PI3K-AKT pathway as observed with acquisition of doxorubicin resistance in both doxorubicin-resistant breast cancer cell line and leukemic cell line (Hui et al., 2008a,b; Chen et al., 2010). Is the nuclear localization of FOXO3a in the Doxorubicin-resistant cells is also caused by Ser-7 phosphorylation or is there any other mechanism? Is the Ser-7-phosphorylated FOXO3a functionally active in mediating the drug resistance? More importantly, can this marker be used in predicting doxorubicin response of tumor cells? Such application would be clinically useful in guiding the choice of chemotherapeutic regime.

The authors report they confirm that p38 mediates FOXO3a phosphorylation on Ser-7 *in vivo*, by the use of a phospho-specific antibody, anti-P-FOXO3a-(Ser-7) which they had generated and validated. This *in vivo* study however was performed on MCF7 breast cancer cell lines that were co-transfected with pCMV-FLAG-FOXO3a and increasing amounts of pEGFP-p38α. It could be argued that this disagrees with the conventional definition of *in vivo* study, as “*in vivo*” generally refers to using whole, living organisms whilst cell line study is usually considered as “*in vitro*.” Regardless, this article presented solid evidences to support the definitive conclusion that p38 directly phosphorylated FOXO3a at Ser-7 and provides new insights into the regulation of FOXO3a. Furthermore, it has provided a nuclear FOXO3a marker, anti-P-FOXO3a-(Ser-7) which has high potential for application on clinical samples, thus implicating some very interesting and worthwhile further research directions.

## References

[B1] Biggs W. H. Meisenhelder J. Hunter T. Cavenee W. K. Arden K. C. (1999). Protein kinase B/Akt-mediated phosphorylation promotes nuclear exclusion of the winged helix transcription factor FKHR1. Proc. Natl. Acad. Sci. U.S.A. 96, 7421–7426 10.1073/pnas.96.13.7421 10377430 PMC22101

[B2] Chen J. Gomes A. R. Monteiro L. J. Wong S. Y. Wu L. H. Ng T. T. (2010). Constitutively nuclear FOXO3a localization predicts poor survival and promotes Akt phosphorylation in breast cancer. PLoS ONE 5:e12293 10.1371/journal.pone.0012293 20808831 PMC2924889

[B3] Gomes A. R. Brosens J. J. Lam E. W. (2008). Resist or die: FOXO transcription factors determine the cellular response to chemotherapy. Cell Cycle 7, 3133–3136 10.4161/cc.7.20.6920 18927504

[B4] Ho K. K. McGuire V. A. Koo C. Y. Muir K. W. de Olano N. Maifoshie E. (2012). Phosphorylation of FOXO3a on Ser-7 by p38 promotes its nuclear localization in response to doxorubicin. J. Biol. Chem. 287, 1545–1555 10.1074/jbc.M111.284224 22128155 PMC3256863

[B5] Hui R. C. Francis R. E. Guest S. K. Costa J. R. Gomes A. R. Myatt S. S. (2008a). Doxorubicin activates FOXO3a to induce the expression of multidrug resistance gene ABCB1 (MDR1) in K562 leukemic cells. Mol. Cancer Ther. 7, 670–678 10.1158/1535-7163.MCT-07-0397 18347152

[B6] Hui R. C. Gomes A. R. Constantinidou D. Costa J. R. Karadedou C. T. Fernandez de Mattos S. (2008b). The forkhead transcription factor FOXO3a increases phosphoinositide-3 kinase/Akt activity in drug-resistant leukemic cells through induction of PIK3CA expression. Mol. Cell. Biol. 28, 5886–5898 10.1128/MCB.01265-07 18644865 PMC2547013

[B7] Paradis S. Ruvkun G. (1998). *Caenorhabditis elegans* Akt/PKB transduces insulin receptor-like signals from AGE-1 PI3 kinase to the DAF-16 transcription factor. Genes Dev. 12, 2488–2498 10.1101/gad.12.16.2488 9716402 PMC317081

[B8] Sunters A. Madureira P. A. Pomeranz K. M. Aubert M. Brosens J. J. Cook S. J. (2006). Paclitaxel-induced nuclear translocation of FOXO3a in breast cancer cells is mediated by c-Jun NH2-terminal kinase and Akt. Cancer Res. 66, 212–220 10.1158/0008-5472.CAN-05-1997 16397234

[B9] Wilson M. S. Brosens J. J. Schwenen H. D. Lam E. W. (2011). FOXO and FOXM1 in cancer: the FOXO-FOXM1 axis shapes the outcome of cancer chemotherapy. Curr. Drug Targets 12, 1256–1266 10.2174/138945011796150244 21443467

[B10] Yang J. Y. Hung M. C. (2009). A new fork for clinical application: targeting forkhead transcription factors in cancer. Clin. Cancer Res. 15, 752–757 10.1158/1078-0432.CCR-08-0124 19188143 PMC2676228

[B11] Yang J. Y. Zong C. S. Xia W. Yamaguchi H. Ding Q. Xie X. (2008). ERK promotes tumorigenesis by inhibiting FOXO3a via MDM2-mediated degradation. Nat. Cell Biol. 10, 138–148 10.1038/ncb1676 18204439 PMC2376808

